# Routine Vaccination During Pregnancy Among People Living With HIV in the United States

**DOI:** 10.1001/jamanetworkopen.2024.9531

**Published:** 2024-05-02

**Authors:** Saba Berhie, Deborah Kacanek, Jessica Lee, Jennifer Jao, Kathleen Powis, Liz Salomon, Danish Siddiqui, Lynn M. Yee

**Affiliations:** 1 Division of Maternal-Fetal Medicine, Department of Obstetrics and Gynecology, Northwestern University Feinberg School of Medicine, Chicago, Illinois; 2 Division of Maternal-Fetal Medicine, Department of Obstetrics and Gynecology, Brigham and Women’s Hospital, Boston, Massachusetts; 3 Center for Biostatistics in AIDS Research, Department of Biostatistics, Harvard T.H. Chan School of Public Health, Boston, Massachusetts; 4 Division of Infectious Diseases, Department of Pediatrics, Northwestern University Feinberg School of Medicine, Chicago, Illinois; 5 Departments of Internal Medicine and Pediatrics, Massachusetts General Hospital, Boston, Massachusetts; 6 Department of Immunology and Infectious Diseases, Harvard T.H. Chan School of Public Health, Boston, Massachusetts; 7 Department of Epidemiology, Harvard T.H. Chan School of Public Health, Boston, Massachusetts; 8 American University of Integrative Sciences, St Michael, Barbados

## Abstract

**Importance:**

Pregnancy represents a window of opportunity for vaccination due to established maternal and fetal benefits of vaccination. Little is known about receipt of routinely recommended vaccines in pregnancy, specifically tetanus, diphtheria, plus acellular pertussis (Tdap) and influenza, among pregnant people living with HIV (PLHIV).

**Objective:**

To estimate prevalence of vaccination receipt among pregnant people with HIV (PLHIV) and identify demographic and clinical characteristics associated with vaccination.

**Design, Setting, and Participants:**

This multicenter cohort study included women participating in Women’s Health Study (WHS) of the Surveillance Monitoring for ART Toxicities (SMARTT) Study of the Pediatric HIV/AIDS Cohort Study. The network has been enrolling pregnant PLHIV at 22 US sites since 2007. Participants for this study enrolled between December 2017 and July 2019. Data analysis was conducted from October 2021 to March 2022.

**Exposure:**

Data on vaccination in pregnancy were collected through medical record abstraction.

**Main Outcomes and Measures:**

Vaccination receipt was defined as Tdap vaccination received at less than 36 weeks’ gestation and influenza vaccination at any gestational age, based on current guidelines. Log-binomial and modified Poisson regression models with generalized estimating equations were fit to identify factors associated with successful receipt of (1) Tdap, (2) influenza, and (3) both vaccinations.

**Results:**

A total of 310 pregnancies among 278 people participating in the WHS were included (mean [SD] age, 29.5 [6.1] years; 220 [71%] Black, 77 [25%] Hispanic, and 77 [25%] race and ethnicity other than Black; 64 [21%] with perinatally acquired HIV). Less than one-third of pregnancies were vaccinated as recommended (Tdap, 32.6% [95% CI, 27.4%-38.1%]; influenza, 31.6% [95% CI, 26.5%-37.1%]; both, 22.6% [95% CI, 18.0%-27.6%]). People living with perinatally acquired HIV, those who did not identify as Black, or those who were multiparous had adjusted risk ratios (aRRs) less than 1, while older PLHIV had aRRs greater than 1, but these differences did not reach statistical significance (perinatally acquired HIV: adjusted risk ratio [aRR], 0.46; 95% CI, 0.21-1.02; race other than Black: aRR, 0.53; 95% CI, 0.26-1.08; multiparous: aRR, 0.59; 95% CI, 0.35-1.00; age 24-29 years: aRR, 2.03; 95% CI, 0.92-4.48).

**Conclusions and Relevance:**

In this diverse, multicenter cohort of pregnant PLHIV, receipt of recommended vaccinations was low. Identifying and addressing barriers to vaccination receipt is urgently needed for pregnant people with HIV.

## Key Points

**Question:**

What is the prevalence of routine vaccination among pregnant people living with HIV?

**Findings:**

In this cohort study of 310 pregnancies among 278 people living with HIV, less than one-third of participants received recommended vaccines in pregnancy: influenza and tetanus, diphtheria, and pertussis. Lower numerical rates of vaccination were observed among multiparous participants and those with perinatally acquired HIV, but the differences did not reach statistical significance.

**Meaning:**

Given the importance of antenatal vaccination for maternal-child health, the data in this study suggest that pregnant people living with HIV have urgent need for clinical and public health interventions to improve vaccine receipt.

## Teaser

This cohort study estimates the prevalence of vaccination receipt among pregnant people with HIV and identifies demographic and clinical characteristics associated with vaccination.

## Introduction

Vaccinations are a critical component of routine prenatal care. Three vaccines are currently recommended in pregnancy by the American College of Obstetricians and Gynecologists (ACOG) and the US Centers for Disease Control and Prevention (CDC): the combined tetanus, diphtheria, and acellular pertussis (Tdap); influenza; and COVID-19 vaccines.^1,2,3^ Pregnant people are at increased risk of influenza-related morbidity and mortality, which has motivated the recommendation for antenatal influenza vaccination with the secondary benefit of neonatal protection.^1,4,5,6^ Additionally, after 27 weeks’ gestation there is fetal benefit with maternal Tdap vaccination, regardless of the recency of prior maternal Tdap vaccination.^2^ The antenatal Tdap vaccine acts as a protective bridge to the neonate’s first dose of the pertussis vaccine at 2 months of life.^6^ Lastly, severe outcomes have been observed among pregnant people who acquire SARS-CoV-2 infection,^7,8,9,10^ and with demonstrated safety and efficacy of COVID-19 vaccinations, vaccination during pregnancy is recommended for all individuals.^11,12,13,14,15,16,17^

Despite extensive and conclusive data on the safety and efficacy of influenza and Tdap vaccines during pregnancy, vaccination in the United States is not universal.^18^ A nationwide survey of pregnant people conducted between 2019 and 2020 found only 69.2% of pregnant persons reported receiving an influenza vaccination and 56.6% received Tdap. Of this cohort, only 40.3% received both recommended vaccines during pregnancy.^19^ Retrospective data from an academic medical center in Chicago found higher, but still suboptimal, rates of vaccine uptake in pregnancy, at 70% for influenza and 87% for Tdap.^20^ Data on COVID-19 vaccination also suggest low receipt in pregnancy; as of July 2021, only 21.8% of pregnant people identified in the CDC vaccine safety database received 1 or more doses of a COVID-19 vaccine during pregnancy.^21,22^

Vaccination recommendations for pregnant people living with HIV (PLHIV) mirror those for pregnant people without HIV, with some augmentation (ie, consideration of hepatitis A, hepatitis B, pneumococcal, and meningococcal vaccination, if indicated) due to their immunocompromised status. However, there are few studies on antenatal vaccination for PLHIV. One study from Atlanta assessed vaccination in pregnancy and found that PLHIV were less likely to be vaccinated against either influenza (4.8% vs 10.3%) or the influenza/Tdap combination (39.7% vs 43.3%) compared with peers without HIV.^23^ Data on other vaccinations among PLHIV are limited but suggest receipt of other vaccines, such as the human papillomavirus (HPV) vaccine, which is recommended to be administered post partum, is similarly low.^24^ Influenza vaccination rates for the general population of people living with HIV is also suboptimal, with data from a large health system in California from 2013 to 2018 revealing influenza vaccination uptake at 65% to 69% for PLHIV.^25^ Given the paucity of data on the topic of routine vaccination in PLHIV, our objective was to estimate the prevalence of and identify factors associated with receiving influenza and/or Tdap vaccinations among pregnant PLHIV participating in a large multisite prospective cohort in the United States.

## Methods

The Surveillance Monitoring for ART Toxicities (SMARTT) study, conducted by the Pediatric HIV/AIDS Cohort Study (PHACS) network, has been enrolling pregnant PLHIV at 22 US sites since 2007. The SMARTT study is designed to evaluate the safety of antiretroviral medications taken during pregnancy by PLHIV by evaluating pregnancy outcomes and the health of their children, who are living with perinatal HIV exposure but uninfected. Between December 1, 2017, and July 31, 2019, SMARTT PLHIV who were either pregnant or had an enrolled child younger than 5 years of age were invited to participate in the Women’s Health Study (WHS), a nested substudy within SMARTT that examined the health of pregnant and nonpregnant PLHIV. The WHS consisted of expanded data collection on pregnancies and maternal health. While new data collection in the WHS took place between 2017 and 2019, the design of the study allowed for earlier medical record abstraction on all pregnancies of PLHIV enrolled in SMARTT. The institutional review board at each site reviewed and approved the study. Participants provided written informed consent prior to their participation in SMARTT. This report follows the STROBE reporting guidelines for observational studies.

The study population for this analysis was derived from participants in the WHS (Figure). The analysis for this study began in October 2021 and was completed in March 2022. Eligibility was restricted to people with available vaccination data in pregnancy and who delivered on or after March 15, 2015, through February 28, 2020. The primary outcome was vaccination receipt during pregnancy, which was assessed through medical record data abstraction; vaccination case report forms that were retuned without indication of vaccination were considered nonvaccination. The following vaccination outcomes were considered: Tdap at 27 weeks of gestation or greater, influenza vaccine received at any time during pregnancy, and both Tdap and influenza vaccines with appropriate timing. ACOG recommends receiving Tdap prior to 36 weeks’ gestation, but as this may not always be clinically feasible and there may still be fetal benefit with later administration, for this analysis, any individual with Tdap receipt between 27 weeks’ gestation and delivery was considered to have received Tdap.^2^ Due to the long influenza season and accordingly wide availability of influenza vaccination at most clinical sites (typically from September to May), we considered all individuals to be eligible for influenza vaccination during pregnancy, as pregnancy periods of all individuals who experienced a live birth would have overlapped with this time. Receipt of the COVID-19 vaccine was not analyzed as vaccine data for this analysis were collected through February 2020.

**Figure.**
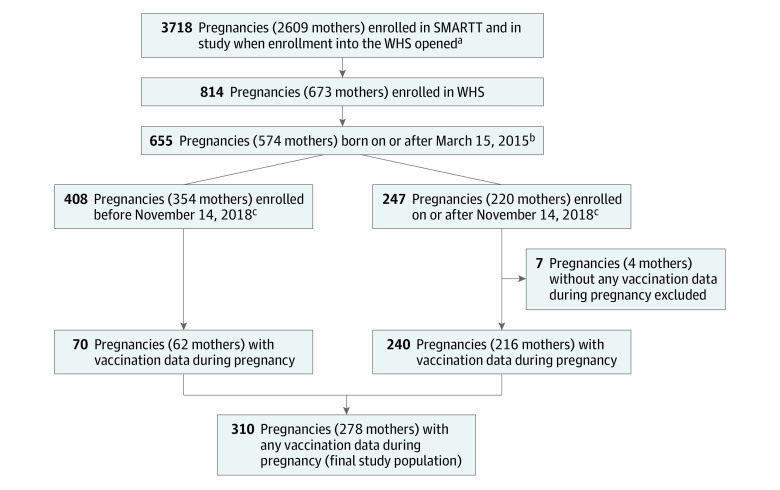
Study Population Selection SMARTT indicates Surveillance Monitoring for ART Toxicities. ^a^Only pregnancies and mothers who were still in the study on December 1, 2017 (when the Women’s Health Study [WHS] opened), were included in the count. ^b^Data on hypertension (a clinical covariate of interest) were collected starting in 2015. ^c^Vaccination data were collected beginning November 14, 2018. Mothers enrolled before November 14, 2018, could still have vaccination data if there was delay between the enrollment visit and when the study staff actually abstracted the information or if the mother was co-enrolled in AMP Up and had medical record abstracted data available.

Variables of interest included demographic characteristics, including age at conception, year of conception, self-identified race (American Indian, Asian, Black, Native Hawaiian or Other Pacific Islander, White, and and unknown), ethnicity (Hispanic or Latinx, non-Hispanic or -Latinx), highest education level, annual household income, and clinical characteristics, including mode of HIV acquisition (perinatally acquired HIV vs non–perinatally acquired HIV), timing of prenatal care initiation (first trimester vs second or third trimester), parity, comorbidities (presence of asthma, diabetes, or chronic hypertension), and any substance use during pregnancy. Race was categorized as Black, race other than Black, or unknown given that 71% of the study population was Black. Substance use was defined as use of tobacco, alcohol, cannabis, sedatives, methamphetamine, cocaine, heroin, MDMA, ketamine, opium, methadone, inhalants, or hallucinogens.

### Statistical Analysis

For each vaccine outcome, the proportions of pregnancies with vaccination receipt and exact 95% CIs under a binomial distribution were estimated. Distributions of the demographic and clinical characteristics were summarized with proportions for categorical variables and means for continuous variables. Characteristics of eligible pregnancies are reported overall and for each of the 3 vaccination outcomes.

Univariable and multivariable log-binomial regression models were fit for each of the vaccine outcomes with generalized estimating equations (GEEs) to account for correlation between multiple pregnancies per participant. An exchangeable correlation structure was assumed for univariable models. For multivariable models, modified Poisson regression was used with an independent correlation structure, a simpler structure, to assure model convergence. Three types of multivariable models were fit: model 1 included only demographic characteristics, model 2 included only clinical characteristics, and model 3 included all demographic and clinical characteristics. The quasi-likelihood under the independence model criterion (QIC) statistics were compared across the 3 models to assess model fit.^26^ Model 3’s QIC demonstrated best fit and is presented in the results as the adjusted estimates. All statistical tests were 2-sided and conducted with an α of less than .05. All analyses were performed using SAS software, version 9.4 (SAS Institute).

## Results

Of 3718 pregnancies from 2609 PLHIV enrolled in SMARTT, 673 PLHIV participated in the WHS substudy, contributing 814 pregnancies. Within this group, 310 pregnancies (among 278 PLHIV) met eligibility criteria for this analysis (Figure). eTable 1 in Supplement 1 describes the study population in contrast to those who did not meet eligibility criteria. Participants with unknown characteristics have been excluded from the percentage calculation for the respective characteristic. Pregnancies included were more frequently of people who were high school graduates (238 of 310 [79%] vs 346 of 504 [70%]), nulliparous (86 [28%] vs 68 [17%]), were living with perinatally acquired HIV (64 [21%] vs 62 [12%]), and had lower frequency of comorbidities (79 [27%] vs 114 [33%]) in contrast to those excluded.

Characteristics of the 310 pregnancies of 278 individuals are summarized in Table 1. Among all pregnancies, the mean (SD) age at conception was 29.5 (6.1) years. Overall, 220 patients (71%) were Black; 77 (25%) were Hispanic, and 77 (25%) identified as a race other than Black; 238 (77%) were experienced by those with at least a high school education. A total of 64 pregnancies (21%) were experienced by participants with perinatally-acquired HIV, 217 (70%) were experienced by individuals who were multiparous, and 215 (69%) were experienced by individuals who had initiated prenatal care by the end of the first trimester of pregnancy. eTable 2 in Supplement 1 describes characteristics of the sample by the type of vaccine received.

**Table 1.** Characteristics of 310 Eligible Pregnancies Among People Living With HIV

Characteristic	Pregnancies, No. (%) (N = 310)
Maternal age at conception, y	
<24	66 (21.3)
24-29	106 (34.2)
30-35	83 (26.8)
≥36	55 (17.7)
Year of conception	
2014-2015	72 (23.2)
2016-2017	112 (36.1)
2018-2019	126 (40.6)
Race	
Black	220 (71.0)
Other	77 (24.8)
Unknown	13 (4.2)
Ethnicity	
Hispanic or Latinx	77 (24.8)
Not Hispanic or Latinx	233 (75.2)
Highest education level	
Less than high school	65 (21.0)
High school or GED	127 (41.0)
More than high school	111 (35.8)
Unknown	7 (2.3)
Annual household income, $	
≤10 000	122 (39.4)
10 001-20 000	64 (20.6)
20 001-30 000	46 (14.8)
≥30 001	49 (15.8)
Unknown	29 (9.4)
Mode of HIV acquisition	
PHIV	64 (20.6)
Non-PHIV	245 (79.0)
Unknown	1 (0.3)
Parity	
Nulliparous	86 (27.7)
Multiparous	217 (70.0)
Unknown	7 (2.3)
Timing of initiation of prenatal care	
At conception or first trimester	215 (69.4)
Second or third trimester	81 (26.1)
Unknown	14 (4.5)
Comorbidities^a^	
Yes	79 (25.5)
No	210 (67.7)
Unknown	21 (6.8)
Substance use during pregnancy^b^	
Yes	83 (26.8)
No	221 (71.3)
Unknown	6 (1.9)

Abbreviation: PHIV, perinatally acquired HIV.

^a^ Presence of asthma, diabetes, or chronic hypertension.

^b^ Tobacco, alcohol, marijuana, sedatives (including barbiturates, benzodiazepines, tranquilizers), methamphetamine, cocaine, heroin, MDMA, ketamine, opium, methadone, inhalants, or hallucinogens (including PCP, LSD).

Tdap vaccine was administered in 32.6% (95% CI, 27.4%-38.1%) of the pregnancies, influenza in 31.6% (95% CI, 26.5%-37.1%), and both vaccines in 22.6% (95% CI, 18.0%-27.6%). Vaccination was less frequent in pregnancies of individuals who were younger, multiparous, had low income, or had perinatally acquired HIV (eFigure in Supplement 1).

In univariable regression analyses, the proportion of pregnancies of multiparous PLHIV with influenza vaccine receipt was lower than that among nulliparous PLHIV (Table 2), and this difference persisted in the multivariable model (adjusted risk ratio [aRR], 0.57; 95% CI, 0.39-0.83). Other clinical or demographic factors were not significantly associated with receipt of the influenza vaccine.

**Table 2.** Unadjusted and Adjusted Estimates of Association of Maternal Characteristics With Influenza Vaccination During Pregnancy

Covariate	Unadjusted			Adjusted^a^		
	No.	RR (95% CI)	*P* value	No.	RR (95% CI)	*P* value
Maternal age at conception, y						
<24	66	1 [Reference]	.77	51	1 [Reference]	.24
24-29	106	1.26 (0.79-2.01)		82	1.41 (0.84-2.36)	
30-35	83	1.25 (0.76-2.05)		61	1.76 (1.03-3.02)	
≥36	55	1.18 (0.68-2.04)		42	1.68 (0.89-3.15)	
Year of conception						
2014-2015	72	1 [Reference]	.08	64	1 [Reference]	.21
2016-2017	112	1.44 (0.93-2.22)		87	1.47 (0.89-2.42)	
2018-2019	126	1.62 (1.03-2.53)		85	1.49 (0.88-2.52)	
Maternal race						
Black	220	1 [Reference]	.97	172	1 [Reference]	.74
Other	77	0.99 (0.68-1.44)		64	0.90 (0.50-1.65)	
Maternal ethnicity						
Hispanic or Latino	77	1 [Reference]	.41	53	1 [Reference]	.92
Not Hispanic or Latino	233	1.19 (0.79-1.79)		183	0.97 (0.52-1.80)	
Mother’s education level						
Less than high school	65	1 [Reference]	.55	47	1 [Reference]	.53
High school or GED	127	0.82 (0.55-1.23)		102	0.80 (0.50-1.29)	
More than high school	111	0.79 (0.51-1.20)		87	0.74 (0.44-1.23)	
Annual household income, $						
≤10 000	122	1 [Reference]	.93	91	1 [Reference]	.98
10 001-20 000	64	1.01 (0.67-1.53)		57	0.93 (0.59-1.48)	
20 001-30 000	46	1.01 (0.64-1.60)		44	0.94 (0.55-1.61)	
≥30 001	49	0.87 (0.52-1.45)		44	0.90 (0.50-1.62)	
Maternal mode of HIV acquisition						
Non-PHIV	245	1 [Reference]	.84	184	1 [Reference]	.55
PHIV	64	0.96 (0.62-1.47)		52	0.86 (0.52-1.41)	
Parity						
Nulliparous	86	1 [Reference]	.03	69	1 [Reference]	.004
Multiparous	217	0.69 (0.50-0.96)		167	0.57 (0.39-0.83)	
Timing of initiation of prenatal care						
At conception or first trimester	215	1 [Reference]	.29	170	1 [Reference]	.27
Second or third trimester	81	0.82 (0.56-1.19)		66	0.78 (0.51-1.20)	
Maternal comorbidities^b^						
No	210	1 [Reference]	.80	169	1 [Reference]	.34
Yes	79	1.05 (0.73-1.50)		67	0.82 (0.55-1.23)	
Substance use during pregnancy^c^						
No	221	1 [Reference]	.52	174	1 [Reference]	.23
Yes	83	0.89 (0.62-1.28)		62	0.75 (0.47-1.20)	

Abbreviations: RR, risk ratio; PHIV, perinatally acquired HIV.

^a^ Multivariable model accounts for all factors.

^b^ Presence of asthma, diabetes, or chronic hypertension.

^c^ Tobacco, alcohol, marijuana, sedatives (including barbiturates/benzodiazepines/tranquilizers), methamphetamine, cocaine, heroin, MDMA, ketamine, opium, methadone, inhalants, or hallucinogens (including PCP, LSD).

After adjusting for maternal and clinical characteristics, pregnancy calendar year was associated with Tdap vaccination; the aRR of Tdap vaccination was 1.88 (95% CI, 1.14-3.13) in 2016 to 2017 compared with pregnancies in 2014 to 2015. Our data also suggested pregnancies of PLHIV with perinatally acquired HIV had lower Tdap vaccination compared with those with non–perinatally acquired HIV, but the difference was not statistically significant (aRR, 0.57; 95% CI, 0.31-1.03). Furthermore, pregnancies of multiparous PLHIV had an aRR of less than 1 for Tdap vaccination, but the results were not statistically significant (aRR, 0.71; 95% CI, 0.47-1.07) (Table 3).

**Table 3.** Unadjusted and Adjusted Estimates of Association of Maternal Characteristics With Tdap Vaccination During Pregnancy

Covariate	Unadjusted			Adjusted^a^		
	No.	RR (95% CI)	*P* value	No.	RR (95% CI)	*P* value
Maternal age at conception, y						
<24	66	1 [Reference]	.34	51	1 [Reference]	.19
24-29	106	1.48 (0.95-2.32)		82	1.70 (0.95-3.04)	
30-35	83	1.39 (0.85-2.27)		61	1.86 (0.98-3.51)	
36 and older	55	1.27 (0.73-2.21)		42	1.55 (0.74-3.25)	
Year of conception						
2014-2015	72	1 [Reference]	.10	64	1 [Reference]	.03
2016-2017	112	1.55 (1.02-2.37)		87	1.88 (1.14-3.13)	
2018-2019	126	1.41 (0.90-2.21)		85	1.55 (0.91-2.64)	
Maternal race						
Black	220	1 [Reference]	.85	172	1 [Reference]	.58
Other	77	1.03 (0.73-1.48)		64	0.86 (0.49-1.48)	
Maternal ethnicity						
Hispanic or Latino	77	1 [Reference]	.61	53	1 [Reference]	.24
Not Hispanic or Latino	233	1.10 (0.75-1.63)		183	0.71 (0.41-1.24)	
Mother’s education level						
Less than high school	65	1 [Reference]	.26	47	1 [Reference]	.65
High school or GED	127	0.78 (0.53-1.15)		102	0.95 (0.60-1.50)	
More than high school	111	0.69 (0.45-1.05)		87	0.80 (0.48-1.33)	
Annual household income, $						
≤10 000	122	1 [Reference]	.59	91	1 [Reference]	.48
10 001-20 000	64	1.27 (0.86-1.86)		57	1.38 (0.91-2.07)	
20 001-30 000	46	0.95 (0.58-1.54)		44	1.05 (0.58-1.88)	
≥30 001	49	0.95 (0.57-1.58)		44	1.06 (0.57-1.98)	
Maternal mode of HIV acquisition						
Non-PHIV	245	1 [Reference]	.11	184	1 [Reference]	.06
PHIV	64	0.67 (0.41-1.10)		52	0.57 (0.31-1.03)	
Parity						
Nulliparous	86	1 [Reference]	.29	69	1 [Reference]	.10
Multiparous	217	0.83 (0.60-1.17)		167	0.71 (0.47-1.07)	
Timing of initiation of prenatal care						
At conception or first trimester	215	1 [Reference]	.75	170	1 [Reference]	.86
Second or third trimester	81	1.06 (0.75-1.49)		66	1.03 (0.72-1.48)	
Maternal comorbidities^b^						
No	210	1 [Reference]	.95	169	1 [Reference]	.39
Yes	79	0.99 (0.69-1.42)		67	0.84 (0.56-1.25)	
Substance use during pregnancy^c^						
No	221	1 [Reference]	.88	174	1 [Reference]	.94
Yes	83	0.97 (0.69-1.38)		62	1.02 (0.65-1.59)	

Abbreviations: RR, risk ratio; PHIV, perinatally acquired HIV.

^a^ Multivariable model accounts for all factors.

^b^ Presence of asthma, diabetes, or chronic hypertension.

^c^ Tobacco, alcohol, marijuana, sedatives (including barbiturates/benzodiazepines/tranquilizers), methamphetamine, cocaine, heroin, MDMA, ketamine, opium, methadone, inhalants, or hallucinogens (including PCP, LSD).

Finally, in evaluating receipt of both influenza and Tdap vaccines, pregnancies of multiparous PLHIV had an aRR less than 1 relative to pregnancies of nulliparous PLHIV for both vaccines, but the results were not statistically significant (aRR, 0.59; 95% CI, 0.35-1.00) (Table 4). Pregnancies of PLHIV with perinatally acquired HIV had aRRs less than 1 for receiving both vaccines (aRR, 0.46; 95% CI, 0.21-1.02), as did pregnancies of individuals who did not identify as Black (aRR, 0.53; 95% CI, 0.26-1.08). In contrast, pregnancies of individuals with older age had aRRs greater than 1 for receiving both vaccines; however, confidence intervals were wide (eg, age 24-29 years: aRR, 2.03; 95% CI, 0.92-4.48) (Table 4).

**Table 4.** Unadjusted and Adjusted Estimates of Association of Maternal Characteristics With Both Influenza and Tdap Vaccination During Pregnancy

Covariate	Unadjusted			Adjusted^a^		
	No.	RR (95% CI)	*P* value	No.	RR (95% CI)	*P* value
Maternal age at conception, y						
<24	66	1 [Reference]	.18	51	1 [Reference]	.16
24-29	106	1.80 (0.94-3.45)		82	2.03 (0.92-4.48)	
30-35	83	1.89 (0.95-3.75)		61	2.43 (1.05-5.61)	
36 and older	55	1.70 (0.80-3.59)		42	2.21 (0.89-5.49)	
Year of conception						
2014-2015	72	1 [Reference]	.11	64	1 [Reference]	.19
2016-2017	112	1.71 (0.94-3.11)		87	1.78 (0.91-3.47)	
2018-2019	126	1.74 (0.95-3.20)		85	1.47 (0.74-2.92)	
Maternal race						
Black	220	1 [Reference]	.36	172	1 [Reference]	.08
Other	77	0.79 (0.47-1.31)		64	0.53 (0.26-1.08)	
Maternal ethnicity						
Hispanic or Latino	77	1 [Reference]	.33	53	1 [Reference]	.25
Not Hispanic or Latino	233	1.30 (0.77-2.20)		183	0.66 (0.33-1.33)	
Mother’s education level						
Less than high school	65	1 [Reference]	.36	47	1 [Reference]	.61
High school or GED	127	0.67 (0.40-1.12)		102	0.80 (0.42-1.50)	
More than high school	111	0.72 (0.43-1.23)		87	0.71 (0.36-1.38)	
Annual household income, $						
≤10 000	122	1 [Reference]	.78	91	1 [Reference]	.73
10 001-20 000	64	1.06 (0.63-1.80)		57	1.20 (0.69-2.09)	
20 001-30 000	46	0.75 (0.38-1.49)		44	0.80 (0.35-1.82)	
≥30 001	49	0.96 (0.52-1.79)		44	1.15 (0.56-2.39)	
Maternal mode of HIV acquisition						
Non-PHIV	245	1 [Reference]	.07	184	1 [Reference]	.06
PHIV	64	0.53 (0.27-1.05)		52	0.46 (0.21-1.02)	
Parity						
Nulliparous	86	1 [Reference]	.30	69	1 [Reference]	.05
Multiparous	217	0.80 (0.52-1.23)		167	0.59 (0.35-1.00)	
Timing of initiation of prenatal care						
At conception or first trimester	215	1 [Reference]	.42	170	1 [Reference]	.23
Second or third trimester	81	0.82 (0.50-1.33)		66	0.71 (0.41-1.24)	
Maternal comorbidities^b^						
No	210	1 [Reference]	.85	169	1 [Reference]	.25
Yes	79	1.05 (0.66-1.65)		67	0.74 (0.45-1.23)	
Substance use during pregnancy^c^						
No	221	1 [Reference]	.98	174	1 [Reference]	.70
Yes	83	1.01 (0.64-1.60)		62	0.89 (0.50-1.59)	

Abbreviations: RR, risk ratio; GED, general education development; PHIV, perinatally acquired HIV.

^a^ Multivariable model accounts for all factors.

^b^ Presence of asthma, diabetes, or chronic hypertension.

^c^ Tobacco, alcohol, marijuana, sedatives (including barbiturates/benzodiazepines/tranquilizers), methamphetamine, cocaine, heroin, MDMA, ketamine, opium, methadone, inhalants, hallucinogens (including PCP, LSD).

## Discussion

This study is among few of which we are aware to describe the prevalence and correlates of Tdap and influenza vaccination in pregnancy for PLHIV. In this cohort, receipt of Tdap and influenza vaccines in pregnancy was low, with fewer than one-third receiving either vaccination and fewer than one-quarter receiving both vaccines, based on medical records. The relative risk of receiving Tdap or both vaccines was also lower in pregnancies of PLHIV who acquired HIV perinatally than among those acquiring HIV later in life. Additionally, pregnancies of multiparous PLHIV had lower relative risk of receiving the influenza vaccine in pregnancy compared with nulliparous PLHIV, whereas pregnancies of older PLHIV had a greater relative risk of receiving both vaccinations. There were also temporal changes in vaccine receipt seen with higher rates in the latter part of the cohort timeline.

Our results demonstrate low receipt of routine vaccinations in a cohort of pregnant PLHIV. Vaccine uptake in the general population of pregnant people is below public health goals, with reports of vaccination from the CDC’s nationwide survey from 2019 to 2020 showing that 61.2% of pregnant people reported receiving influenza vaccination, 56.6% received Tdap, and 40.3% received both.^27^ The Tdap vaccination has been recommended as standard of care for every pregnant individual by the CDC’s Advisory Committee on Immunization Practices expanded since 2013.^28^ Data from a single institution demonstrated a temporal finding in Tdap vaccination rates: vaccination uptake increased over time, with an increase from 47.4% to 86.1% between 2011 and 2015.^20^ However, our findings suggest an increase between 2014 and 2015 (15.8%) to 2016 and 2017 (40.6%) but then suggests a potential plateau from 2017 onward, as the 2018 and 2019 uptake was stable at 43.9%. Additionally, in this same study by DiTosto et al,^20^ it was hypothesized that the 2013 recommendation for Tdap vaccination in pregnancy may have increased influenza vaccine uptake, as receipt of the influenza vaccine went from 61.2% to 72.0% between 2011 and 2015 in the general pregnant population. A trend toward similar findings was seen in our data, although further work is required.

Like previous studies, we found nulliparity to be associated with higher probability of influenza vaccine receipt, whereas the association of parity with vaccination was less pronounced for Tdap.^20,29^ This difference may be explained by the perceived difference between the Tdap and influenza vaccines. Tdap is counseled as a vaccine for fetal and neonatal benefit while influenza is counseled as a vaccine to prevent maternal as well as neonatal morbidity and mortality. Individuals who are pregnant for the first time may be more hesitant to take on the personal risk of influenza illness than those who have been through a pregnancy. It is important to note that vaccination in pregnancy is not only important for the maternal-fetal dyad, but a person’s decision to decline recommended vaccinations in pregnancy is a predictor for poor childhood vaccine uptake.^30^

In this diverse cohort, the aRRs for receipt of both vaccines were less than 1 among individuals who did not identify as Black (compared with those who were Black), although findings did not achieve statistical significance. This finding contrasts with other studies that observed lower receipt of vaccination during and outside of pregnancy among people identifying as Black.^31^ Additionally, lower vaccination receipt has been documented among people who are Black and/or low-income, who may be less likely to be offered, trust, or accept vaccines such as HPV, influenza, pneumococcal, and COVID-19 compared with individuals who are White or have higher income.^32,33,34,35,36^ A rigorous, multipronged approach to understand the role of social determinants of health with regard to vaccination and address them through policy change and programmatic intervention is urgently needed.^37^

We found that people with perinatally acquired HIV had lower rates of vaccination in pregnancy compared with those who acquired HIV later in life. This finding fits with other data comparing clinical outcomes between these 2 groups. People with perinatally acquired HIV have distinct challenges in the setting of pregnancy and have complex psychosocial, socioeconomic, and medical concerns.^38,39,40,41,42^

Pregnancy is a period of enhanced health care access and engagement, serving as a critical window of opportunity for preventive health interventions such as vaccination. Additionally, pregnant people are at increased risk of morbidity and mortality from infections such as influenza.^18^ Thus, counseling about and receiving antenatal vaccinations are of utmost importance for all pregnant people, especially those with immunocompromising comorbidities like HIV. While some data suggest lower vaccination immunogenicity in PLHIV without optimal control and more advanced disease, no risks have been revealed and vaccination continues to be the standard of care in this population.^43,44,45^ Evidence-based interventions to improve vaccine receipt exist in pediatric and adolescent care, where vaccinations are an integral part of routine care. Successful interventions incorporate a multipronged approach in efforts to improve vaccine uptake. Novel interventions, such as reminder text messages to parents, web-based informational sites, and placing prepopulated orders in clinic records to limit health care professional error, have demonstrated improved outcomes.^46,47^ In the adult population, a randomized clinical trial demonstrated that in a group of predominantly racial and ethnic minority participants, a pneumococcal vaccination video and brochure was associated with increased vaccination rates compared with being shown an informational video alone. This dual information approach was also associated with more documented physician-patient discussion about the vaccine.^48^ However, little such work has extended to pregnant PLHIV.

Our findings build upon prior published literature that demonstrate that people have suboptimal receipt of recommended vaccines in pregnancy and show that pregnant PLHIV are even less likely to receive these recommended vaccinations relative to historical general population estimates. Individuals in this population may also have not been offered a vaccine. Future studies utilizing self-reporting methods and comparing survey research methods with chart abstractions would be beneficial for better comparing our results with those of other studies. Alternatively, it could be that the lower prevalence of vaccination receipt we observed was due to lack of trust in the health care system, which can influence health care utilization for PLHIV. PLHIV are often from communities that have been systematically disenfranchised, exploited, and marginalized and as a result may face barriers to accessing high-quality health care, or have experienced discrimination or been mistreated in other ways during medical encounters, which could increase skepticism of health care systems.^49,50^ Future research on interventions and innovation in counseling and information dissemination is needed among pregnant PLHIV to better understand the reasons for and circumstances surrounding suboptimal receipt of vaccines during pregnancy. Understanding the perspectives of pregnant people with and without HIV regarding vaccination during pregnancy is crucial and can inform successful clinical and social interventions to improve vaccination during pregnancy. Our team is conducting ongoing qualitative work on this issue, aiming to identify targeted programming and interventions capable of improving routine vaccination in pregnancy. Such work also needs to address how strategies that are developed for well-established vaccinations can be rapidly adapted to novel vaccinations, particularly for vulnerable populations. Future work should also consider clinician- and health system–based barriers to receiving recommended vaccinations during pregnancy.

### Strengths and Limitations

One strength of our study is that this is the largest assessment of routine vaccination for pregnant PLHIV to date and includes data from a well-characterized, nationally representative cohort. Our diverse population is also a strength, as pregnant people from underrepresented communities are often not included in research.

However, there are limitations to note. Although participants were enrolled prospectively, the data on vaccination receipt do not offer additional insight beyond whether and when the vaccine was administered; data obtained via medical record abstraction do not detail the reasons for lack of vaccination. Such reasons may include inadequate clinician recommendation for vaccination, participant refusal of vaccination, or barriers to health care access that preclude vaccination. Retrospective data review of medical records may also underestimate vaccination, such as when patients received vaccination in an outside medical system. Despite SMARTT being one of the largest studies of its kind in the United States, our sample size is limited due to the subset of pregnancies with data collection on vaccination, thus restricting power to detect difference by covariates of interest. Additionally, the confidence intervals estimated for the outcomes may be affected by correlation between repeat pregnancies.^51^ Furthermore, the participants who enrolled in the SMARTT cohort may not represent the HIV community with respect to vaccination, as they may feel more comfort and integration with the health care system and potentially have higher vaccination rates compared with those who chose not to participate in SMARTT. Future work should corroborate these results in larger and ongoing cohorts.

## Conclusions

Understanding barriers to and perspectives on routine vaccination for pregnant PLHIV is of vital importance to improve the health outcomes of pregnant people and their infants. Our data demonstrate suboptimal receipt of antenatal vaccines with evidence-based benefit in a cohort of pregnant PLHIV. Clinicians, researchers, and public health systems must identify and evaluate the impact of innovative and impactful strategies, including improved patient-facing messaging, as the current approach is unsuccessful at achieving the clinical and public health goals of widespread antenatal vaccination.

## Notes

Supplement 1. **eFigure.** Prevalence of Vaccine Uptake in Pregnancy Among Persons Living With HIV in SMARTT, by Selected Maternal Characteristics (2015-2019) **eTable 1.** Distribution of Characteristics by Inclusion in Study Population **eTable 2.** Characteristics of Eligible Pregnancies by Type of Vaccination

Supplement 2.
Nonauthor Collaborators

Supplement 3.
Data Sharing Statement
