# Supplementary material for: Routine Vaccination During Pregnancy Among People Living With HIV in the United States
Source: JAMA Netw Open. 2024 May 2;7(5):e249531. doi: 10.1001/jamanetworkopen.2024.9531 (PMC11066702; doi:10.1001/jamanetworkopen.2024.9531)
Supplement: Supplement 1. — eFigure. Prevalence of Vaccine Uptake in Pregnancy Among Persons Living With HIV in SMARTT, by Selected Maternal Characteristics (2015-2019) eTable 1. Distribution of Characteristics by Inclusion in Study Population eTable 2. Characteristics of Eligible Pregnancies by Type of Vaccination [file jamanetwopen-e249531-s001.pdf]

Supplemental Online Content

Berhie S, Kacanek D, Lee J, et al; Pediatric HIV/AIDS Cohort Study. Routine vaccination during pregnancy among people living with HIV in the United States. *JAMA Netw Open.* 2024;7(5):e249531. doi:10.1001/jamanetworkopen.2024.9531

eFigure. Prevalence of Vaccine Uptake in Pregnancy Among Persons Living With HIV in SMARTT, by Selected Maternal Characteristics (2015-2019)

eTable 1. Distribution of Characteristics by Inclusion in Study Population

eTable 2. Characteristics of Eligible Pregnancies by Type of Vaccination

This supplemental material has been provided by the authors to give readers additional information about their work.

© 2024 Berhie S et al. *JAMA Network Open.*

eFigure. Prevalence of Vaccine Uptake in Pregnancy Among Persons Living With HIV in SMARTT, by Selected Maternal Characteristics (2015-2019)

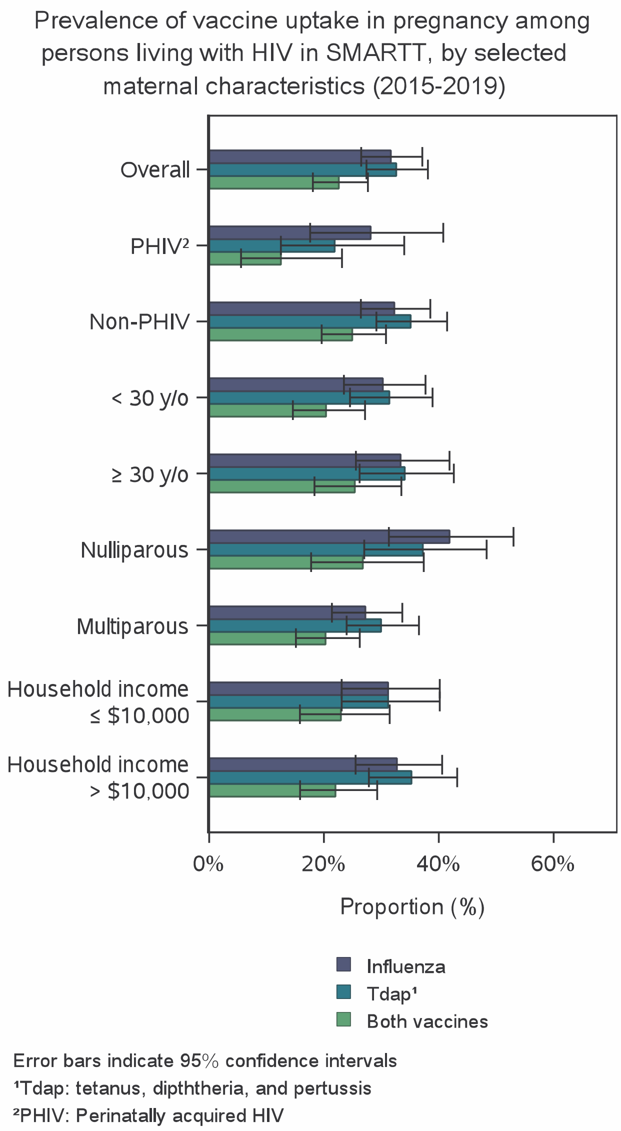

© 2024 Berhie S et al. *JAMA Network Open.*

eTable 1. Distribution of Characteristics by Inclusion in Study Population

Characteristic		Study Population Inclusion Yes (N=310)	No (N=504)	Total (N=814)
Age at conception	Years, mean ± SD	29.5 ± 6.1	29.3 ± 6.0	29.3 ± 6.0
Year of conception	2012/2013	0 (0%)	102 (20%)	102 (13%)
	2014/2015	72 (23%)	186 (37%)	258 (32%)
	2016/2017	112 (36%)	168 (33%)	280 (34%)
	2018/2019	126 (41%)	48 (10%)	174 (21%)
Race	Black	220 (74%)	338 (70%)	558 (72%)
	Non-Black	77 (26%)	143 (30%)	220 (28%)
	Unknown	13	23	36
Ethnicity	Hispanic or Latinx	77 (25%)	149 (30%)	226 (28%)
	Not Hispanic or Latinx	233 (75%)	354 (70%)	587 (72%)
	Unknown	0 (0%)	1 (0%)	1 (0%)
Highest education level[1]	Less than high school	65 (21%)	151 (30%)	216 (27%)
	High school or GED	127 (42%)	179 (36%)	306 (38%)
	More than high school	111 (37%)	167 (34%)	278 (35%)
	Unknown	7	7	14
Annual household income	≤ $10,000	122 (43%)	239 (51%)	361 (48%)
	$10,001 - $20,000	64 (23%)	106 (23%)	170 (23%)
	$20,001 - $30,000	46 (16%)	56 (12%)	102 (14%)
	≥ $30,001	49 (17%)	65 (14%)	114 (15%)
	Unknown	29	38	67
Mode of HIV acquisition	Non-PHIV	245 (79%)	437 (88%)	682 (84%)
	PHIV	64 (21%)	62 (12%)	126 (16%)
	Unknown/Missing	1	5	6
Parity	Nulliparous	86 (28%)	68 (17%)	154 (22%)
	Multiparous	217 (72%)	327 (83%)	544 (78%)
	Unknown	7	109	116
Timing of initiation of prenatal care	At conception or 1st trimester	215 (73%)	370 (77%)	585 (76%)
	2nd or 3rd trimester	81 (27%)	108 (23%)	189 (24%)
	Unknown	14	26	40
Comorbidities[1]	Yes	79 (27%)	114 (33%)	193 (30%)
	No	210 (73%)	231 (67%)	441 (70%)
	Unknown	21	159	180
Substance use during Pregnancy[2]	Yes	83 (27%)	123 (25%)	206 (26%)
	No	221 (73%)	372 (75%)	593 (74%)
	Unknown	6	9	15

SD: standard deviation; GED: general education development; PHIV: perinatally acquired HIV

1. Presence of asthma, diabetes, or chronic hypertension
2. Tobacco, alcohol, marijuana, sedatives (including barbiturates/benzodiazepines/tranquilizers), methamphetamine, cocaine, heroin, MDMA, ketamine, opium, methadone, inhalants, or hallucinogens (including PCP, LSD)

© 2024 Berhie S et al. *JAMA Network Open.*

eTable 2. Characteristics of Eligible Pregnancies by Type of Vaccination

Characteristic		Total (N=310) N (%)	Received Influenza vaccine (N=98) N (%)	Received TDaP[1] vaccine (N=101) N (%)	Received both vaccines (N=70) N (%)
Maternal age at conception (in years)	Mean (SD)[1]	29.5 (6.1)	29.7 (6.0)	29.8 (5.9)	30.3 (5.8)
Maternal age at conception	Under 24	66 (21.3)	18 (18.4)	16 (15.8)	9 (12.9)
	24-29	106 (34.2)	34 (34.7)	38 (37.6)	26 (37.1)
	30-35	83 (26.8)	28 (28.6)	29 (28.7)	22 (31.4)
	36 and older	55 (17.7)	18 (18.4)	18 (17.8)	13 (18.6)
Year of conception	2014/2015	72 (23.2)	15 (15.3)	16 (15.8)	10 (14.3)
	2016/2017	112 (36.1)	38 (38.8)	41 (40.6)	28 (40.0)
	2018/2019	126 (40.6)	45 (45.9)	44 (43.6)	32 (45.7)
Maternal race	Black	220 (71.0)	73 (74.5)	74 (73.3)	55 (78.6)
	Non-Black	77 (24.8)	25 (25.5)	27 (26.7)	15 (21.4)
	Unknown	13 (4.2)			
Maternal ethnicity	Hispanic or Latino	77 (24.8)	21 (21.4)	23 (22.8)	14 (20.0)
	Not Hispanic or Latino	233 (75.2)	77 (78.6)	78 (77.2)	56 (80.0)
Mother's education level[1]	Less than high school	65 (21.0)	24 (24.5)	27 (26.7)	19 (27.1)
	High school or GED	127 (41.0)	38 (38.8)	39 (38.6)	24 (34.3)
	More than high school	111 (35.8)	32 (32.7)	31 (30.7)	23 (32.9)
	Unknown	7 (2.3)	4 (4.1)	4 (4.0)	4 (5.7)
Annual household income	≤ $10,000	122 (39.4)	38 (38.8)	38 (37.6)	28 (40.0)
	$10,001 - $20,000	64 (20.6)	22 (22.4)	26 (25.7)	16 (22.9)
	$20,001 - $30,000	46 (14.8)	16 (16.3)	14 (13.9)	8 (11.4)
	≥ $30,001	49 (15.8)	14 (14.3)	16 (15.8)	11 (15.7)
	Unknown	29 (9.4)	8 (8.2)	7 (6.9)	7 (10.0)
Maternal mode of HIV acquisition[1]	PHIV	64 (20.6)	18 (18.4)	14 (13.9)	8 (11.4)
	Non-PHIV	245 (79.0)	79 (80.6)	86 (85.1)	61 (87.1)
	Unknown	1 (0.3)	1 (1.0)	1 (1.0)	1 (1.4)
Parity	Nulliparous	86 (27.7)	36 (36.7)	32 (31.7)	23 (32.9)
	Multiparous	217 (70.0)	59 (60.2)	65 (64.4)	44 (62.9)
	Unknown	7 (2.3)	3 (3.1)	4 (4.0)	3 (4.3)
Timing of initiation of prenatal care	At conception or 1st trimester	215 (69.4)	70 (71.4)	66 (65.3)	49 (70.0)
	2nd or 3rd trimester	81 (26.1)	23 (23.5)	28 (27.7)	16 (22.9)
	Unknown	14 (4.5)	5 (5.1)	7 (6.9)	5 (7.1)
Maternal comorbidities[2]	Yes	79 (25.5)	26 (26.5)	26 (25.7)	19 (27.1)
	No	210 (67.7)	66 (67.3)	69 (68.3)	48 (68.6)

© 2024 Berhie S et al. *JAMA Network Open.*

Characteristic		Total (N=310) N (%)	Received Influenza vaccine (N=98) N (%)	Received TDaP[1] vaccine (N=101) N (%)	Received both vaccines (N=70) N (%)
	Unknown	21 (6.8)	6 (6.1)	6 (5.9)	3 (4.3)
Substance use during pregnancy[3]	Yes	83 (26.8)	23 (23.5)	26 (25.7)	18 (25.7)
	No	221 (71.3)	72 (73.5)	72 (71.3)	49 (70.0)
	Unknown	6 (1.9)	3 (3.1)	3 (3.0)	3 (4.3)

[1] TDaP: Tetanus, diphtheria, and pertussis vaccine; SD: standard deviation; GED: General education development; PHIV: Perinatally acquired HIV

[2] Presence of asthma, diabetes, or chronic hypertension

[3] Tobacco, alcohol, marijuana, sedatives (including barbiturates/benzodiazepines/tranquilizers),methamphetamine, cocaine, heroin, MDMA, ketamine, opium, methadone, inhalants, hallucinogens (including PCP, LSD)

© 2024 Berhie S et al. *JAMA Network Open.*
