# Supplementary material for: Routine Vaccination During Pregnancy Among People Living With HIV in the United States
Source: JAMA Netw Open. 2024 May 2;7(5):e249531. doi: 10.1001/jamanetworkopen.2024.9531 (PMC11066702; doi:10.1001/jamanetworkopen.2024.9531)
Supplement: Supplement 2. — Nonauthor Collaborators [file jamanetwopen-e249531-s002.pdf]

Supplemental Online Content: Nonauthor Collaborators
*First name, last name, and suffix (if applicable) are required and will appear in PubMed.

*Group Name(s): Pediatric HIV/AIDS Cohort Study

*First Name and Middle Initial(s)	*Last Name	*Suffix (eg, Jr, III)	Academic Degrees	Institution	Location (city, state/province, country)	Role or Contribution, eg, chair, principal investigator	Group (if more than 1 Group listed in the byline) and/or Subgroup (eg, Steering Committee)
Jessica	D'Angelo			Ann & Robert H. Lurie Children’s Hospital of Chicago:			
Margarent	Ann Sanders			Ann & Robert H. Lurie Children’s Hospital of Chicago:			
Malle	Kathleen			Ann & Robert H. Lurie Children’s Hospital of Chicago:			
Mary	Paul			Baylor College of Medicine			
Ruth	Eser-Jose			Baylor College of Medicine			
Chivon	McMullen-Jackson			Baylor College of Medicine			
Lynnette	Harris			Baylor College of Medicine			
Murli	Purswani			BronxCare Health System			
Mahoobullah	Mirza Baig			BronxCare Health System			
Alma	Villegas			BronxCare Health System			
Marvin	Alvarado			BronxCare Health System			
Lisa-Gaye	Robinson			Children's Diagnostic & Treatment Center			
Jawara Dia	Cooley			Children's Diagnostic & Treatment Center			
James	Blood			Children's Diagnostic & Treatment Center			
Patricia	Garvie			Children's Diagnostic & Treatment Center			
William	Borkowsky			New York University School of Medicine			
Nagamah	Deygoo			New York University School of Medicine			
Jennifer	Lewis			New York University School of Medicine			

Supplemental Online Content: Nonauthor Collaborators
*First name, last name, and suffix (if applicable) are required and will appear in PubMed.

***First Name and Middle Initial(s)**	***Last Name**	***Suffix (eg, Jr, III)**	Academic Degrees	Institution	Location (city, state/province, country)	Role or Contribution, eg, chair, principal investigator	Group (if more than 1 Group listed in the byline) and/or Subgroup (eg, Steering Committee)
Arry	Dieudonne			Rutgers - New Jersey Medical School			
Linda	Bettica			Rutgers - New Jersey Medical School			
Juliette	Johnson			Rutgers - New Jersey Medical School			
Karen	Suroweic			Rutgers - New Jersey Medical School			
Katherine	Knapp			St. Jude Children's Research Hospital			
Jamie	Russell-Bell			St. Jude Children's Research Hospital			
Megan	Wilkins			St. Jude Children's Research Hospital			
Stephanie	Love			St. Jude Children's Research Hospital			
Nicolas	Rosario			San Juan Hospital Research Unit/Department of Pediatrics			
Lourdes	Angeli-Nieves			San Juan Hospital Research Unit/Department of Pediatrics			
Vivian	Olivera			San Juan Hospital Research Unit/Department of Pediatrics			
Stephan	Kohlhoff			SUNY Downstate Medical Center			
Ava	Dennie			SUNY Downstate Medical Center			
Jean	Kaye			SUNY Downstate Medical Center			
Jenny	Wallier			SUNY Downstate Medical Center			
Karen	Craig			Tulane University School of Medicine			
Russell	Van Dyke			Tulane University School of Medicine			

Supplemental Online Content: Nonauthor Collaborators

*First name, last name, and suffix (if applicable) are required and will appear in PubMed.

***First Name and Middle Initial(s)**	***Last Name**	***Suffix (eg, Jr, III)**	Academic Degrees	Institution	Location (city, state/province, country)	Role or Contribution, eg, chair, principal investigator	Group (if more than 1 Group listed in the byline) and/or Subgroup (eg, Steering Committee)
Patricia	Sirois			Tulane University School of Medicine			
Cecelia	Hutto			University of Alabama, Birmingham			
Paige	Hickman			University of Alabama, Birmingham			
Julie	Huldtquist			University of Alabama, Birmingham			
Dan	Marullo			University of Alabama, Birmingham			
Stephen A.	Spector			University of California, San Diego			
Veronica	Figueroa			University of California, San Diego			
Megan	Loughran			University of California, San Diego			
Sharon	Nichols			University of California, San Diego			
Elizabeth	McFarland			University of Colorado, Denver			
Christine	Kwon			University of Colorado, Denver			
Carrie	Glenny			University of Colorado, Denver			
Jennifer	Englund			University of Colorado, Denver			
Mobeen	Rathore			University of Florida			
Saniyyah	Mahmoudi			University of Florida			
Sarah	El-Hassan			University of Florida			
Jamilah	Tejan			University of Florida			
Karen	Hayani			University of Illinois, Chicago			
Lourdes	Richardson			University of Illinois, Chicago			
Renee	Smith			University of Illinois, Chicago			
Alina	Miller			University of Illinois, Chicago			
Gwendolyn	Scott			University of Miami			
Gustavo	Gil Garcia			University of Miami			
Gabriel	Fernandez			University of Miami			
Anai	Cuadra			University of Miami			
Toni	Frederick			Keck Medicine of the University of Southern California			

Supplemental Online Content: Nonauthor Collaborators
*First name, last name, and suffix (if applicable) are required and will appear in PubMed.

***First Name and Middle Initial(s)**	***Last Name**	***Suffix (eg, Jr, III)**	Academic Degrees	Institution	Location (city, state/province, country)	Role or Contribution, eg, chair, principal investigator	Group (if more than 1 Group listed in the byline) and/or Subgroup (eg, Steering Committee)
Mariam	Davtyan			Keck Medicine of the University of Southern California			
Guadalupe	Morales-Avendano			Keck Medicine of the University of Southern California			
Zoe M.	Rodriguez			University of Puerto Rico School of Medicine			
Lizmarie	Torres			University of Puerto Rico School of Medicine			
Nydia	Scalley			University of Puerto Rico School of Medicine			
