# Supplementary material for: Routine Vaccination During Pregnancy Among People Living With HIV in the United States
Source: JAMA Netw Open. 2024 May 2;7(5):e249531. doi: 10.1001/jamanetworkopen.2024.9531 (PMC11066702; doi:10.1001/jamanetworkopen.2024.9531)
Supplement: Supplement 3. — Data Sharing Statement [file jamanetwopen-e249531-s003.pdf]

Data Sharing Statement

Berhie. Routine Vaccination During Pregnancy Among People Living With HIV in the United States. *JAMA Netw Open*. Published May 02, 2024. doi:10.1001/jamanetworkopen.2024.9531

Data
Data available: No
